# Evaluation of Prediction-Oriented Model Selection Metrics for Extended Redundancy Analysis

**DOI:** 10.3389/fpsyg.2022.821897

**Published:** 2022-04-11

**Authors:** Sunmee Kim, Heungsun Hwang

**Affiliations:** ^1^Department of Psychology, University of Manitoba, Winnipeg, MB, Canada; ^2^Department of Psychology, McGill University, Montreal, QC, Canada

**Keywords:** out-of-sample model evaluation, predictive performance analysis, cross-validation, bootstrap, extended redundancy analysis

## Abstract

Extended redundancy analysis (ERA) is a statistical method that relates multiple sets of predictors to response variables. In ERA, the conventional approach of model evaluation tends to overestimate the performance of a model since the performance is assessed using the same sample used for model development. To avoid the overly optimistic assessment, we introduce a new model evaluation approach for ERA, which utilizes computer-intensive resampling methods to assess how well a model performs on unseen data. Specifically, we suggest several new model evaluation metrics for ERA that compute a model’s performance on out-of-sample data, i.e., data not used for model development. Although considerable work has been done in machine learning and statistics to examine the utility of cross-validation and bootstrap variants for assessing such out-of-sample predictive performance, to date, no research has been carried out in the context of ERA. We use simulated and real data examples to compare the proposed model evaluation approach with the conventional one. Results show the conventional approach always favor more complex ERA models, thereby failing to prevent the problem of overfitting in model selection. Conversely, the proposed approach can select the true ERA model among many mis-specified (i.e., underfitted and overfitted) models.

## Introduction

Extended redundancy analysis (ERA; Takane and Hwang, 2005) is a statistical modeling framework that relates multiple sets of predictors to response variables. In ERA, a component is extracted from each set of predictors in such a way that it accounts for the maximum variation of response variables. ERA provides several benefits in terms of interpretability and predictability, especially when many predictors need to be studied in a regression problem. For example, ERA provides a parsimonious interpretation of directional relationships using the extracted components whose number is much smaller than that of the original predictors. Also, using domain-specific knowledge concerning which predictors are to be put together can improve the interpretability of the extracted components. Moreover, ERA allows researchers to generate predictive models because it searches for components that maximize predictive accuracy, without having to eliminate any predictors of interest to avoid multicollinearity. Indeed, the practical usefulness of ERA lies in its predictive nature; ERA has been well blended with many statistical techniques for prediction problems, e.g., regularizations (Hwang et al., 2015; Lee et al., 2019; Kim et al., 2020) and a tree-based supervised learning method (Kim and Hwang, 2021).

The present study concerns model evaluation in ERA. How to assess a model’s performance is critical for model comparison and model selection. One existing method is to calculate FIT (Takane and Hwang, 2005), an *R*^2^-type overall goodness-of-fit (GOF) metric for ERA. Several other GOF metrics for parametric ERA, such as AIC_ERA_ and BIC_ERA_ (DeSarbo et al., 2015), are also available, which are computed based on penalized-likelihood criteria taking model complexity into account. A model’s GOF based on these metrics provides diagnostic information that helps explain model performance by quantifying the degree of similarity between observed and predicted values. However, all of these conventional metrics represent “in-sample” model evaluation, i.e., the same dataset is used both to construct the model and to assess its performance. This naturally leads to an overly optimistic view of a model’s performance: the more closely we fit the model to the training sample—a set of data used to estimate parameters, the better it will perform when being evaluated on the same sample. This is a well-known statistical phenomenon called “optimism” (Efron, 1983; Efron and Tibshirani, 1997; Hastie et al., 2009, Chapter 7).

When researchers are interested in predicting important health or behavioral outcomes to the benefit of the broader population, relying on such an “optimistic” view based on in-sample model evaluation is not ideal because it provides little information about the model’s performance on “out-of-sample”—data that are not used for model building. For example, in studies on cognitive impairment in older adults (Choi and Jin, 2018; Na, 2019), patient responses to treatments for depression (Cuijpers et al., 2013), or user response patterns in online advertising (Zhang et al., 2016), the goal of model evaluation is to select a prediction model that can best assist practitioners with their decision making in unseen cases (e.g., treatment recommendation for new patients). To develop such prediction models that can generalize beyond the current sample, it would be better to assess a model’s performance based on out-of-samples.

Thus, in this paper, we introduce several new model evaluation metrics for ERA, each of which aims to quantify how well a model performs in out-of-sample data. But before discussing the out-of-sample metrics, we also investigate the degree of optimism in the existing in-sample model evaluation based on a simulation study. Hastie et al. (2009, Chapter 7) discussed, in a general regression problem, the optimism of in-sample model evaluation decreases linearly as the training sample size increases but increases with model complexity. Thus, in the simulation study, we examine how the degree of optimism is affected by different training sample sizes and the number of ERA parameters under several model mis-specification conditions.

We then illustrate a new model evaluation framework for ERA which aims to avoid the overly optimistic assessment in the conventional in-sample metrics. Specifically, we apply various sample-reuse or resampling methods, such as cross-validation (CV; Stone, 1974; Geisser, 1975) and the bootstrap (Efron, 1979, 1983), to assess out-of-sample model performance. The basic idea behind this framework is to avoid the optimism by using non-overlapping data for model development and evaluation, which in turn provides a more accurate estimate of model performance in a new sample (Hastie et al., 2009, Chapter 7; Shmueli, 2010). Although considerable work has been done on the use of CV and bootstrap variants for out-of-sample model assessment in various prediction models (e.g., Steyerberg et al., 2001; Molinaro et al., 2005; Smith et al., 2014; Austin and Steyerberg, 2017), to date, no research has applied these general tools to ERA. Thus, we formulate various out-of-sample prediction error estimators for ERA based on (1) *c*-fold CV (*c* = 3, 5, and 10), (2) leave-one-out CV (LOOCV), (3) out-of-bag (OOB) bootstrap, (4) 0.632 bootstrap, and (5) 0.632+ bootstrap, and carry out a simulation to examine their relative behaviors under different simulation conditions.

The remainder of this paper is structured as follows. First, we briefly recapitulate the ERA model and provide a formulation of a prediction error estimator for ERA. We then describe the use of the abovementioned resampling methods for out-of-sample model evaluation in ERA. Next, we present the results of a series of simulation studies comparing the conventional in-sample metrics and new out-of-sample prediction error estimators. In all simulations, mis-specified (i.e., underfitted and overfitted) models are considered to examine the behavior of each estimator. The empirical usefulness of the proposed out-of-sample model evaluation is illustrated using a real-world dataset. Finally, we summarize the findings, provide a guideline for practitioners on which resampling approach should be favored under which conditions, and discuss the limitation of the study.

## Materials and Methods

### Extended Redundancy Analysis

Let *y_i_* denote the *i*th value of the response variable (*i* = 1,  ..., *N*). Assume that there are *K* different sets of predictors, each of which consists of *P_k_* predictors (*k =* 1,  ..., *K*). Let *x_ikp_* denote the *i*th value of the *p*th variable in the *k*th predictor set (*p* = 1,  ..., *P_k_*) and **x***_i_* = (*x*_*i*11_,  ..., *x_ikp_*) denote a 1 by *P* vector of predictors for the *i*th observation, where $P=\overset{K}{\underset{k=1}{\sum }}P_{k}$. Let *w_kp_* denote a component weight assigned to *x_ikp_* and **w***_k_* =${\left(w_{k1},...,w_{kP_{k}}\right)}^{\prime }$denote a *P_k_* by 1 vector of component weights in the *k*th predictor set. Let $f_{ik}=\overset{P_{k}}{\underset{p=1}{\sum }}x_{ikp}w_{kp}$ denote the *i*th component score of the *k*th component, which is the sum of weighted predictors for the *i*th observation in the *k*th predictor set. Let *b_k_* denote the regression coefficient relating the *k*th component to the response variable. Let *e_i_* denote an error term for *y_i_*. We assume that all predictors are standardized with zero means and unit variances (Takane and Hwang, 2005).

The ERA model (Takane and Hwang, 2005) is then expressed as,


(1) $$\begin{array}{l} y_{i}=\overset{K}{\underset{k=1}{\sum }}\left[\overset{P_{k}}{\underset{p=1}{\sum }}x_{ikp}w_{kp}\right]\;b_{k}+e_{i}=x_{i}^{\prime }\text{Wb}+e_{i}\\ \;\;\;=\overset{K}{\underset{k=1}{\sum }}f_{ik}b_{k}+e_{i}=f_{i}b+e_{i}, \end{array}$$

where $W=\left[\begin{array}{ccc} w_{1} & & 0\\ & \ddots & \\ 0 & & w_{k} \end{array}\right]$, $f_{i}=\left(f_{i1},\cdots ,f_{iK}\right)$, and $b={\left(b_{1},\cdots ,b_{K}\right)}^{\prime }$.

This can also be expressed as,


(2) y=XWb+e=Fb+e

where **y** is an *N* by 1 vector of response variables, **X** is an *N* by *P* matrix of predictors, **W** is a *P* by *K* matrix of component weights, **F** is a *N* by *K* matrix of regression coefficients, and **e** is an *N* by 1 vector of errors. For identification, a standardization constraint is imposed on F such that diag(**F**’**F**) = *N***I**, where diag(∙) denotes a diagonal matrix with the elements of **F**’**F** on its diagonal. As shown in (1), each set of predictors reduces to a single component, which in turn influences the response variable. The component weight *w_kp_* shows the contribution of each predictor to obtaining its component as in data reduction methods such as principal component analysis or canonical correlation analysis, whereas the regression coefficient *b_k_* signifies the effect of each component on the response variable as in linear regression. In this regard, ERA carries out data reduction and linear regression simultaneously.

**Figure 1:**
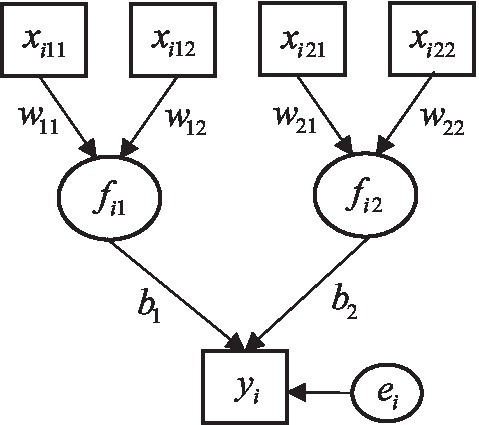
A prototypical ERA model. Square boxes indicate observed predictor and response variables. Circles represent components and an error term.


Figure 1 displays an example of the ERA model, where each component is associated with two predictors (*P_1_* = *P*_2_ = 2) and a response variable is influenced by two components (*K* = 2). For this example, the **W** and **b** are given as,

$$W=\left(\begin{array}{l} w_{11}\;\;\;\;0\\ \begin{array}{cc} w_{12} & 0 \end{array}\\ \begin{array}{cc} 0 & w_{21} \end{array}\\ \begin{array}{cc} 0 & w_{22} \end{array} \end{array}\right)\;\mathrm{and}\;b=\left(\begin{array}{c} b_{1}\\ b_{2} \end{array}\right)$$

The ERA model contains two sets of parameters—component weights (**W**) and regression coefficients (**b**). These unknown parameters are estimated by minimizing the following objective function:


(3) $$\phi =\mathrm{SS}\left(y-XWb\right),$$

with respect to **W** and **b**, subject to the constraint diag(**F**’**F**) = *N***I**, where SS(**a**) = **a**’**a**, i.e., the sum of squares for all the elements of a vector **a**. An alternating least-squares (ALS) algorithm (de Leeuw et al., 1976) was developed to minimize the objective function (Takane and Hwang, 2005). Refer to Takane and Hwang (2005) for a more detailed description of the parameter estimation algorithm.

To assess the performance of ERA models, an overall GOF metric, called FIT (Takane and Hwang, 2005), can be calculated to evaluate how well a given ERA model explains the variance of the response variable:


(4) $$\mathrm{FIT}=1-\frac{\phi }{\mathrm{SS}\left(y\right)}$$

The values of FIT range from 0 to 1, and larger values indicate more explained variance of the response variable. The limitation of using FIT in conventional ERA will be discussed later but note that it only informs how well a model fits to the training set, thus being of little utility when assessing a model’s prediction capability.

### Assessment of Predictive Performance

Consider a continuous response variable *Y* that is related to a predictor matrix **X** by a prediction model *h*: **X** → *Y*. Then, $\hat{h}\left(X\right)$ denotes the predicted responses estimated from the observed training set *T* = {$\left(x_{1}^{\prime },y_{1}\right)$, ⋯,$\left(x_{i}^{\prime },y_{i}\right)$, ⋯,$\left(x_{N}^{\prime },y_{N}\right)$}. We assume that the observations in *T* are random samples from a distribution *H*. Let denote $L\left(Y,\hat{h}\left(X\right)\right)$ a loss function for measuring errors between *Y* and $\hat{h}\left(X\right)$. For example, in many regression-based models, a common choice for a continuous *Y* is the squared-error loss, i.e., $L\left(Y,\hat{h}\left(X\right)\right)={\left(Y-\hat{h}\left(X\right)\right)}^{2}$. The term “loss” in mathematical optimization is used to describe how much a model is losing compared to having made perfect predictions. Thus, a loss function is a measure of how good a model does in terms of being able to predict the expected responses. Note that, as shown in (3), ERA defines its objective function in terms of the mean squared-error loss and seeks to minimize it over all possible parameter values, which shows its predictive nature.

To quantify the overall predictive performance of an ERA model, root mean square error (RMSE),


(5) $$\mathrm{RMSE}=\sqrt{\frac{1}{N}\sum_{i=1}^{N}{\left(y_{i}-\hat{h}\left(x_{i}\right)\right)}^{2}}$$

is a reasonable choice because ERA parameters are estimated to minimize the mean squared-error loss, as discussed above. This metric can range from 0 to ∞ and is negatively oriented scores, which means lower values are better. Also, it is indifferent to the direction of errors since the errors are squared before they are averaged.

### Estimating Generalization Error Using Resampling Methods

In predictive modeling, we wish to obtain a model that not only performs well on the training data, but also on independent unseen data. Thus, it is important to understand how to estimate the true error rate of a model when it is used to predict the future responses. Let $\left(x_{0}^{\prime },y_{0}\right)$ is a new independent test sample randomly drawn from *H*. The *true test error* or *generalization error* (Efron, 1983; Efron and Tibshirani, 1997; Hastie et al., 2009, Chapter 7) is the prediction error for $\left(x_{0}^{\prime },y_{0}\right)$,


(6) $${\mathrm{Err}}_{T}=E_{\left(x_{0}^{\prime },y_{0}\right)}\left[L\left(y_{0},\hat{h}\left(x_{0}\right)\right)|Τ\right]$$

Note that, in (6), only $\left(x_{0}^{\prime },y_{0}\right)$ is random with *T* being fixed, meaning that the true test error refers to the conditional error for the particular training set *T*. In practice, it is more amenable to estimate a model’s prediction error as the expectation of ${\mathrm{Err}}_{T}$ (Efron, 1983; Efron and Tibshirani, 1997; Hastie et al., 2009, Chapter 7),


(7) $$\mathrm{Err}=E\left[{\mathrm{Err}}_{T}\right]=E\left[L\left(Y,\hat{h}\left(X\right)\right)\right]$$

where everything random is averaged over. In many machine learning applications, where a large independent test set is available, the goal of model selection is to find a model that gives the minimum expected test error in (7).

In the absence of a large independent test set, the simplest way to estimate ${\mathrm{Err}}_{T}$ is to use the *training error* or *apparent error*, defined by the average loss over the training data,


(8) $${\mathrm{Err}}_{\mathrm{Train}}=\frac{1}{N}\overset{N}{\underset{i=1}{\sum }}L\left(y_{i},\hat{h}\left(x_{i}\right)\right)$$

As discussed previously, ${\mathrm{Err}}_{\mathrm{Train}}$ is typically smaller than ${\mathrm{Err}}_{T}$, i.e., ${\mathrm{Err}}_{\mathrm{Train}}$ tends to be biased downward as an estimate of ${\mathrm{Err}}_{T}$ because the same observations are used twice, both for fitting $\hat{h}\left(X\right)$ and for evaluating the prediction error of $\hat{h}\left(X\right)$. Note that, in conventional ERA, model evaluation is based only on this apparent performance.

A straightforward approach for correcting the inherent optimism in ${\mathrm{Err}}_{\mathrm{Train}}$ is to randomly split the observed data *T* in two parts: one for developing the model (*learning set*) and the other for measuring its predictive performance (*validation set*). With this split-sample approach, model performance can be assessed on independent data not used for model development. There are, however, two criticisms of this procedure. First, it is inefficient when the size of *T* is small, owing to its reduction of the size of both learning and validation sets. Second, high variability in the estimated predictive performance can be introduced because of its reliance on a single split of *T*.

Various computer-intensive resampling methods can be employed to avoid these limitations of the split-sample approach. Let ${\bar{\mathrm{Err}}}_{\left(\mathrm{Resampling Method}\right)}$ denote the estimated generalization error (7) of an ERA model, which is formulated as the averaged predictive performance on validation data, where the predictive performance is computed as described in (5) and validation data are generated based on a resampling method. Using this out-of-sample prediction error estimator, we can now define the optimal model as the one gives the lowest ${\bar{\mathrm{Err}}}_{\left(\mathrm{Resampling Method}\right)}$ (i.e., the smallest prediction error on validation data) in model selection. Key references on the use of different resampling methods (for validation data creation) include Efron (1983), Breiman and Spector (1992), and Efron and Tibshirani (1997).

*c*-fold CV is one of the most preferred methods used to generate validation data. This method randomly assigns *N* observations to one of *c* partitions such that the partitions are of nearly equal size. Subsequently, the learning set contains all but one of the partitions which is labeled the validation set. After fitting a model to the learning set, the prediction error (RMSE) of the fitted model is computed using the validation set. We then repeat this procedure for all *c* folds and average the prediction errors, resulting in the *c*-fold CV estimate of prediction error: ${\bar{\mathrm{Err}}}_{\left(\mathrm{cv,}c\right)}$. LOOCV is the most extreme case of *c*-fold CV, where the number of folds equals the number of observations (i.e., *c* = *N*) and each observation is individually assigned to the validation set.

Another popular approach is based on the bootstrap (Efron, 1979). Efron (1983) proposed and compared several bootstrap variants for the assessment of a model’s predictive performance, which are generally referred to as *out-of-bag* (OOB) estimators in the statistics and machine learning literature. Calculating the OOB prediction error begins with bootstrap sampling. Let *B* be the number of bootstrap replications. For each draw, a bootstrap sample contains only 63.2% of the original data on average (referred to as *in-bag sample*) due to the sampling with replacement. The prediction error is assessed on the remaining 37% of the data (OOB data) for each bootstrap draw and subsequently averaged over the *B* iterations, resulting in the OOB estimate of prediction error: ${\bar{\mathrm{Err}}}_{\left(\mathrm{OOB}\right)}$.

There are two more variations of the OOB estimator: 0.632 estimator (Efron, 1983) and 0.632+ estimators (Efron and Tibshirani, 1997). Both aim to correct the underestimated ${\mathrm{Err}}_{\mathrm{Train}}$ as a weighted combination of ${\mathrm{Err}}_{\mathrm{Train}}$ and ${\bar{\mathrm{Err}}}_{\left(\mathrm{OOB}\right)}$ such that *ω*·${\mathrm{Err}}_{\mathrm{Train}}$+ (1-*ω*)·${\bar{\mathrm{Err}}}_{\left(\mathrm{OOB}\right)}$. The value of *ω* is fixed as 0.632 for the 0.632 estimator, whereas *ω* is determined based on the so-called “no-information error rate” for the 0.632+ estimator (Efron and Tibshirani, 1997). In brief, the weight is dependent on the relative amount of overfitting coefficient *R*: *ω* = 0.632/(1–0.368·*R*). The relative overfitting *R* is large when the difference between ${\mathrm{Err}}_{\mathrm{Train}}$ and ${\bar{\mathrm{Err}}}_{\left(\mathrm{OOB}\right)}$ is relatively large. In this case, *R* and *ω* approach 1, indicating that the estimated prediction error is largely based on ${\bar{\mathrm{Err}}}_{\left(\mathrm{OOB}\right)}$. When the overfitting is small, *R* approaches 0 and *ω* 0.632, resulting in similarity between the 0.632 and 0.632+ estimators. In this paper, we also consider these two bootstrap variants for ERA: ${\bar{\mathrm{Err}}}_{\left(.632\right)}$ and ${\bar{\mathrm{Err}}}_{\left(.632+\right)}$.

## Simulation Study

We conducted a simulation study to examine the behaviors of the abovementioned in-sample and out-of-sample prediction error estimators for ERA. Specifically, under different model mis-specification conditions, we investigated the degree of optimism in ${\mathrm{Err}}_{\mathrm{Train}}$ in terms of (4) and (5) and compared the differences between the resampling methods in the estimation of ${\mathrm{Err}}_{T}$: ${\bar{\mathrm{Err}}}_{\left(\mathrm{cv}\right)}$, ${\bar{\mathrm{Err}}}_{\left(\mathrm{LOOCV}\right)}$, ${\bar{\mathrm{Err}}}_{\left(\mathrm{OOB}\right)}$, ${\bar{\mathrm{Err}}}_{\left(.632\right)}$, and ${\bar{\mathrm{Err}}}_{\left(.632+\right)}$. We particularly focused on the influence of sample size and model complexity. For this, we considered five different sample sizes (*N* = 50, 100, 200, 500, 1,000) for training data (*T*) and varied the number of predictors per component (*N_p_* = 2, 4, 6, 8).

For data generation, we specified an ERA model that was composed of two components (*K* = 2) and a response variable (see Figure 1). We fixed the first regression coefficient *b*_1_ to 0.3, and the variance explained by two components *R*^2^ to 0.4, which in turn resulted in the value of the second regression coefficient, *b*_2_ = 0.56. We assumed no correlation between the components. Each component was linked to *N_p_* predictors, where each component weight, *w_kp_*, was randomly sampled from a uniform distribution between 0.8 and 1. Following the data generation approach of Becker et al. (2013), the variance-covariance matrix of the predictor and response variables, Σ, was obtained based on the ERA parameters described above. We generated 1,000 datasets from a multivariate normal distribution with zero means and Σ for each combination of sample sizes (*N*) and the number of predictors per component (*N_p_*).

For each simulation scenario, we considered three different conditions of model mis-specification and investigated if the true (data generating) model was chosen using the various out-of-sample prediction error estimators. The three mis-specified models and the true model were as follow: (1) under-specified model, f0:$y_{i}=b_{1}f_{i1}+e_{i}$, (2) correctly specified model (i.e., data generating model), f1:$y_{i}=b_{1}f_{i1}+b_{2}f_{i2}+e_{i}$, (3) over-specified model with a component interaction term, f2:$y_{i}=b_{1}f_{i1}+b_{2}f_{i2}+b_{3}\left(f_{i1}\cdot f_{i2}\right)+e_{i}$, and (4) over-specified model with interaction and quadratic terms, f3:$y_{i}=b_{1}f_{i1}+b_{2}f_{i2}+b_{3}\left(f_{i1}\cdot f_{i2}\right)+b_{4}\left(f_{i1}{}^{2}\right)+b_{5}\left(f_{i2}{}^{2}\right)+e_{i}$.

All data generation and computations were carried out using the R system for statistical computing (Version 3.4). We wrote an R code to implement ERA and to compute the various in-sample and out-of-sample prediction error estimators for ERA. We used the R package “mlr” (Bischl et al., 2016) to create random samples generated by *c*-fold CV, LOOCV, OOB, 0.632, and 0.632+ bootstrap. See Data and Code Availability for details regarding the code used for the simulation.

### Optimism in In-Sample Metrics

**Figure 2:**
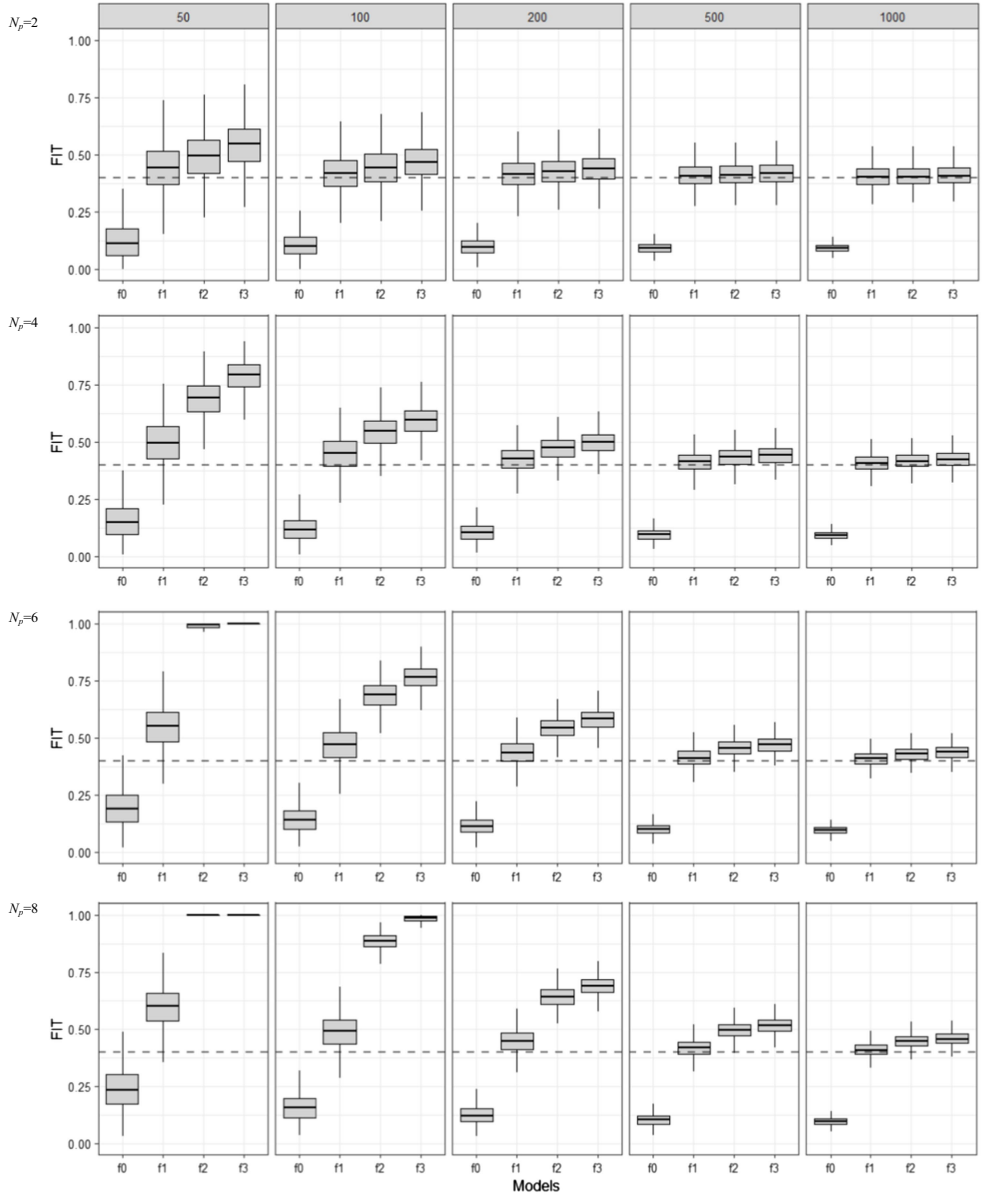
Behavior of in-sample FIT values as the training set sample size (*N* = 50, 100, 200, 500, 1,000) and model complexity (*Np* = 2, 4, 6, 8) are varied for different model mis-specification conditions (f0, f1, f2, and f3). f1 is the true model, f0 is an under-specified model, and f2 and f3 are over-specified models.

Figure 2 shows the training set or apparent FIT values (i.e., in-sample model performance measured by FIT) for four different ERA models (f0, …, f3), including the true and three mis-specified ones, in relation to the sample size (*N*) and the number of indicators per component (*N_p_*). Boxplots were constructed to show the distribution of estimated FIT values over 1,000 repetitions. For each simulation condition, we also estimated the Err estimate in (7)—which was computed using 1,000 independent test sets (of size *N* each)—and added it as a dotted line, so that we can easily view the bias and variability of FIT, as a model performance estimator for ${\mathrm{Err}}_{T}$.

As the boxplots show, when sample size was large and the number of predictors per component was small, the median apparent performance of FIT (centers of boxplots) approached the true test error (dotted lines). FIT’s variability (each boxplot’s length) also decreased in such conditions. However, the true model (f1) was never selected by the FIT, because the overfitted models (f2 and f3) had higher FIT values under all simulation conditions. In addition, the median apparent performance for f1 was always above the dotted line, showing FIT’s optimism as an estimate of ${\mathrm{Err}}_{T}$, as was theoretically expected. For the overfitted models, the apparent performance of FIT tended to reach its maximum value (i.e., 1) rapidly even when sample size was small and the number of predictors per component was large, indicating that the optimism in the apparent FIT metric can be of particular problem when overfitting happens.

**Figure 3:**
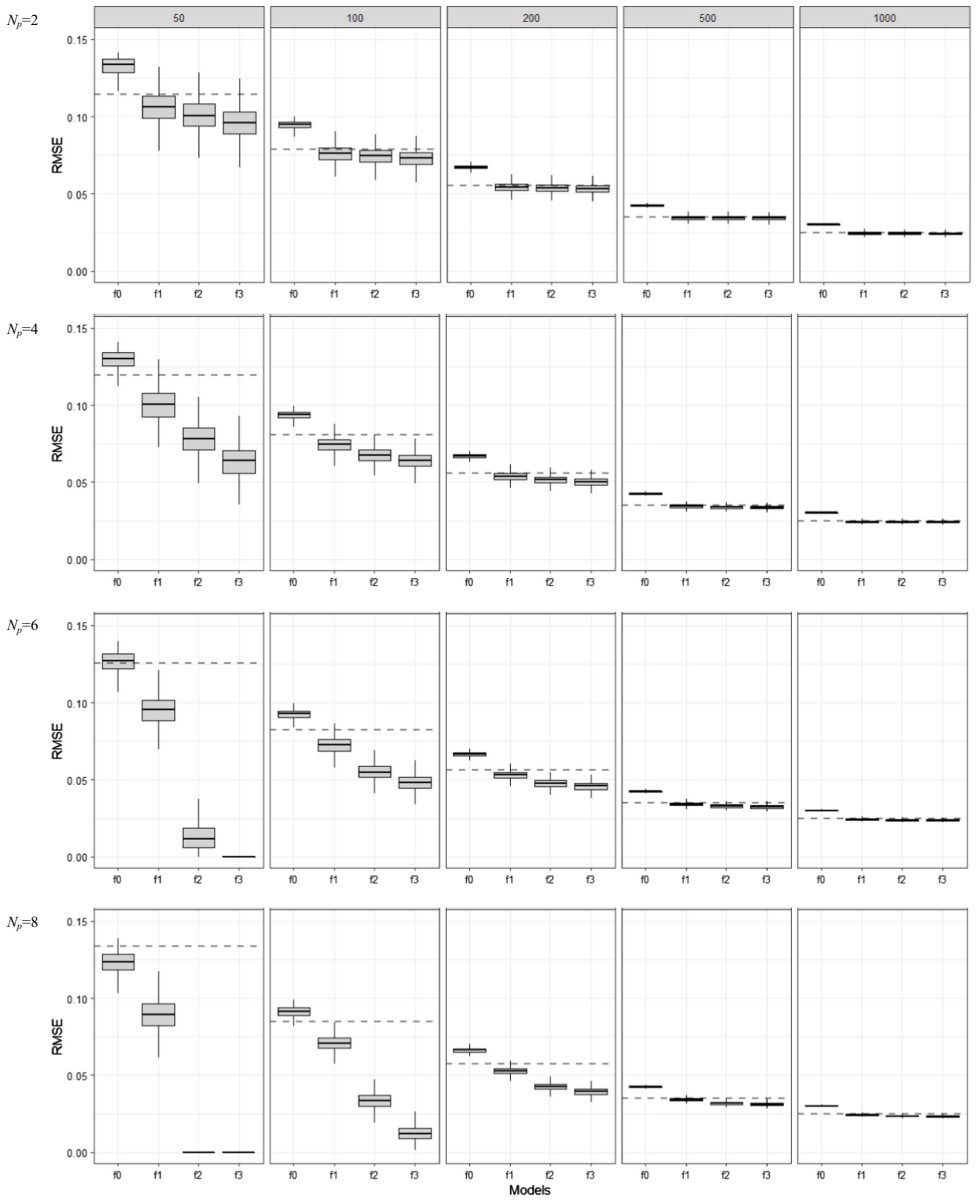
Behavior of in-sample RMSE values as the training set sample size (*N* = 50, 100, 200, 500, 1,000) and model complexity (*Np* = 2, 4, 6, 8) are varied for different model mis-specification conditions (f0, f1, f2, and f3). f1 is the true model, f0 is an under-specified model, and f2 and f3 are over-specified models.

In Figure 3, boxplots show the in-sample model performance measured by RMSE in (5). The differences between Figure 2 and Figure 3 are minimal in terms of the optimism of in-sample model assessment: the apparent RMSE values were clearly estimated below the dotted lines, indicating overly underestimated prediction error. However, RMSE showed less variability than FIT: the difference in the variabilities of apparent RMSE values for f0, f1, f2, and f3 became very minimal when the sample size was larger than 50 (*N* ≥ 100).

### Behavior of Out-of-Sample Estimators

In this section, all simulation results are discussed but only a limited number of figures are displayed because the differences in the error estimators across the resampling methods were negligible as the sample size increased, e.g., *N* > 100. The full compilation of simulation results is archived on the first author’s GitHub, see Data and Code Availability for more details.

**Figure 4:**
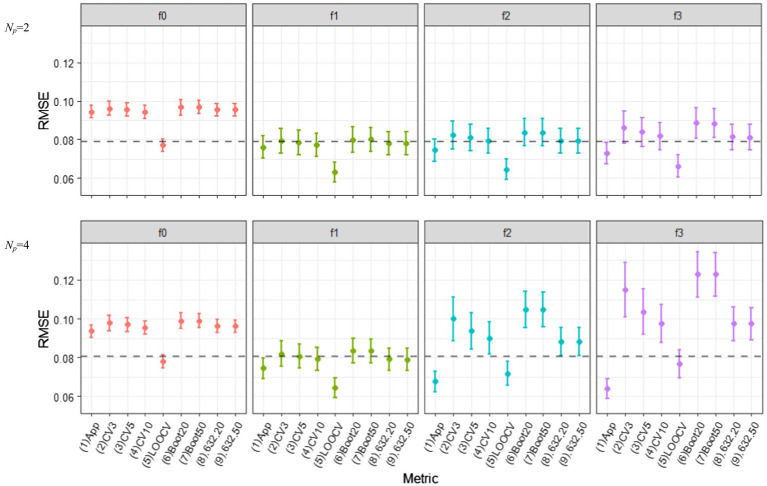
Behavior of out-of-sample prediction error estimators based on various resampling methods, as the model complexity (*Np* = 2 and 4) are varied different conditions of model mis-specifications (f0, f1, f2, and f3). The training sample size is *N* = 100.

Figure 4 displays the behavior of various out-of-sample prediction error estimators based on different resampling methods for the correctly and incorrectly specified ERA models (f0, f1, f2, and f3) when *N* = 100. The figures in the top panels show the results of *N_p_* = 2, where those in the bottom portray the results obtained when *N_p_* = 4. We obtained the results for (1) *c*-fold CV estimators with *c* = 3, 5, and 10 (i.e., ${\bar{\mathrm{Err}}}_{\left(\mathrm{cv,}3\right)}$, ${\bar{\mathrm{Err}}}_{\left(\mathrm{cv,}5\right)}$, ${\bar{\mathrm{Err}}}_{\left(\mathrm{cv,}10\right)}$), (2) LOOCV estimator (${\bar{\mathrm{Err}}}_{\left(\mathrm{LOOCV}\right)}$), (3) the regular OOB bootstrap estimators with *B* = 20, 50, and 100 (${\bar{\mathrm{Err}}}_{\left(\mathrm{OOB,}B=20\right)}$, ${\bar{\mathrm{Err}}}_{\left(\mathrm{OOB,}B=50\right)}$, ${\bar{\mathrm{Err}}}_{\left(\mathrm{OOB,}B=100\right)}$), (4) the 0.632 estimators with *B* = 20, 50, and 100 (${\bar{\mathrm{Err}}}_{\left(.632,B=20\right)}$, ${\bar{\mathrm{Err}}}_{\left(.632,B=50\right)}$, ${\bar{\mathrm{Err}}}_{\left(.632,B=100\right)}$), and (5) the 0.632+ estimators with *B* = 20, 50, and 100 (${\bar{\mathrm{Err}}}_{\left(.632+,B=20\right)}$, ${\bar{\mathrm{Err}}}_{\left(.632+,B=50\right)}$, ${\bar{\mathrm{Err}}}_{\left(.632+,B=100\right)}$). We also computed the apparent RMSE (i.e., in-sample RMSE) and displayed it with other prediction error estimators to see if the optimism remained. This is denoted by “(1) App” in the figure. As there was no noticeable difference between the 0.632 and 0.632+ estimators in all simulation conditions, the results of the 0.632+ estimators are not included in Figure 4. The results obtained from the OOB bootstrap and 0.632 estimators with *B* = 100 are not displayed as well because there was minimal improvement over those with *B* = 50. In the figure, each error bar represents one standard deviation of the expected value of the estimated errors over 1,000 repetitions. Each dotted line indicates the Err estimate in (7) which is estimated over 1,000 simulated independent test sets (of size *N* each).

Most noticeably, all out-of-sample prediction error estimators resulted in the smallest error for f1, on average; thus, the true ERA model was always selected across all simulation conditions. In this true model condition, all of the resampling estimators—except the LOOCV—successfully corrected the optimism in the apparent error estimator: They reported larger values than the underestimated in-sample RSME [“(1) App” in the figure], and at the same time, located closer to the dotted lines. They also exhibited smaller variabilities, compared to those in the over-specified conditions (i.e., f2 and f3). In all sample size and model complexity conditions, the LOOCV estimator for f1 had the lowest variability but showed noticeable downward bias. This is possible because each learning set in the LOOCV procedure is very similar to the full observed data *T* (Efron and Tibshirani, 1997). The *c*-fold CV estimators had smaller biases than other bootstrap variants, regardless of the number of folds, but the difference among the resampling methods was marginal.

When overfitting occurred (f2 and f3), however, the CV estimators overestimated the true prediction error with a high variability when the number of folds was small (*c* = 3 and 5). Similarly, the OOB bootstrap estimators showed large upward biases, but such bias was substantially reduced in the 0.632 and 0.632+ estimators (The advantage of increasing *B* from 20 to 50 was minimal in all bootstrap variant estimators). The variability of both 0.632 and 0.632+ estimators were smaller than that of 10-fold CV. Unlike simulation results in which various regression-based models were considered (e.g., Efron and Tibshirani, 1997; Steyerberg et al., 2001), 0.632+ did not outperform LOOCV in the context of ERA, particularly in small samples and with many predictors.

## Empirical Application

We examined the utility of the proposed out-of-sample model evaluation metrics with a real data application. We used the same ERA model considered in Kim and Hwang (2021) for the analysis of nicotine dependence in the United States. Their model considered the effects of three components of 12 predictors—early exposure to substances (*f*_1_), mental health (*f*_2_), and socioeconomic status (SES, *f*_3_)—on nicotine dependence among American adults. Table 1 shows which component is associated with which predictors. The authors applied the ERA model to the National Survey on Drug Use and Health (NSDUH) data collected in 2012, but for this empirical study, we utilized new data from more recent survey years of NSDUH, 2016–2019 (U.S. Department of Health and Human Services et al., 2017, 2018, 2019, 2020). Table 1 presents a description of the variables included, as well as their summary statistics. As shown in the table, the number of respondents (*N*) varied across survey years, from 6,697 to 7,477. Particularly, the data collected in 2019 (*N* = 6,697) were used as an independent test set for assessing the generalization performance of a model.

**Table 1:** Description of variables and summary statistics for the NSDUH data (2016 ~ 2019).

Variable Names	Measures (Range or Categories)	2016	2017	2018	2019
(Sample Size)		7,477	7,244	6,835	6,697
Response Variable					
Nicotine dependence	Average score over 17 items of the Nicotine Dependence Syndrome Scale (1–5)	2.6(2.0, 3.1)	2.6(2.0, 3.1)	2.6(2.0, 3.0)	2.6(2.1, 3.1)
Predictor Variables					
F_1_: Substance initiation age					
Cigarette	Age of first cigarette use	15.2(13.0, 17.0)	15.2(13.0, 17.0)	15.3(13.0, 17.0)	15.2(13.0, 17.0)
Alcohol	Age of first alcohol use	15.5(14.0, 17.0)	15.5(14.0, 17.0)	15.5(14.0, 17.0)	15.5(14.0, 17.0)
Marijuana	Age of first marijuana use	16.2(14.0, 18.0)	16.2(14.0, 18.0)	16.3(14.0, 18.0)	16.3(14.0, 18.0)
F_2_: Mental health status					
Distress level	Nonspecific psychological distress scale (K6) score	3.4(0.0, 6.0)	3.5(0.0, 6.0)	3.6(0.0, 6.0)	3.9(0.0, 7.0)
Impairment	Daily functional impairment due to problems with emotions, nerves, or mental health	1.7(0.0, 3.0)	1.8(0.0, 3.0)	1.8(0.0, 3.0)	2.0(0.0, 4.0)
Suicidal thought	Serious thoughts of suicide in the past year (Yes = 1/No = 0)	Yes%: 10.1	Yes%: 11.2	Yes%: 11.5	Yes%: 12.2
Depression	Major depressive episode in the past year (*Y* = 1/*N* = 0)	Yes%: 14.4	Yes%: 15.5	Yes%: 15.3	Yes%: 16.8
F_3_: Socioeconomic status					
Education	5th grade or less (=5), 6th grade (=6), …, Freshman/13th year (=13), Sophomore/Junior (=14), Senior/Grad or more (=15)	8.6(8.0, 9.0)	8.5(8.0, 9.0)	8.6(8.0, 9.0)	8.6(8.0, 9.0)
Insurance	Having any health insurance (Y/N)	Yes%: 83.8	Yes%: 82.8	Yes%: 82.4	Yes%: 81.9
Family income	Less than $10,000 (=1), ~$19,999 (=2), ~$29,999 (=3), …, ~$39,999 (=4), ~$49,999 (=5), …, ~$74,999 (=6), $75,000 or more (=7)	4.3(2.0, 6.0)	4.3(2.0, 6.0)	4.4(2.0, 6.0)	4.4(3.0, 6.0)
Employment Status	Employed (*Y* = 1/*N* = 0)	Yes%: 63.5	Yes%: 62.8	Yes%: 63.3	Yes%: 61.9

For continuous variables, mean, the first quartile, and the third quartile are given.

**Figure 5:**
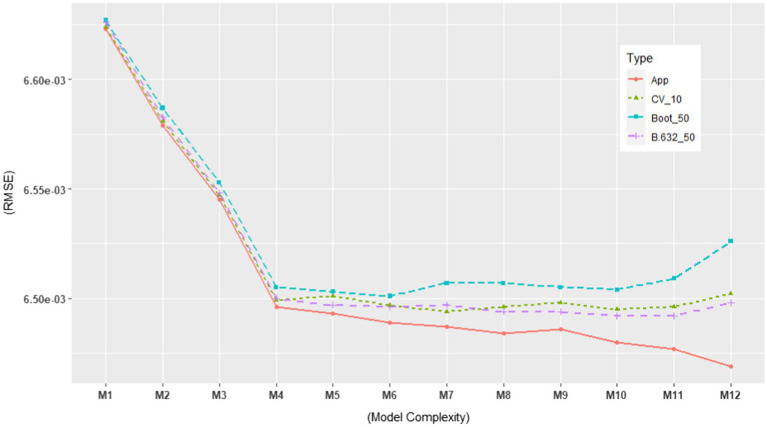
In-sample and out-of-sample RMSE for 12 candidate models using the NSDUH data from 2016 to 2018 (Total *N* = 21,553). The RMSE values are consistent with those in Table 2.

**Figure 6:**
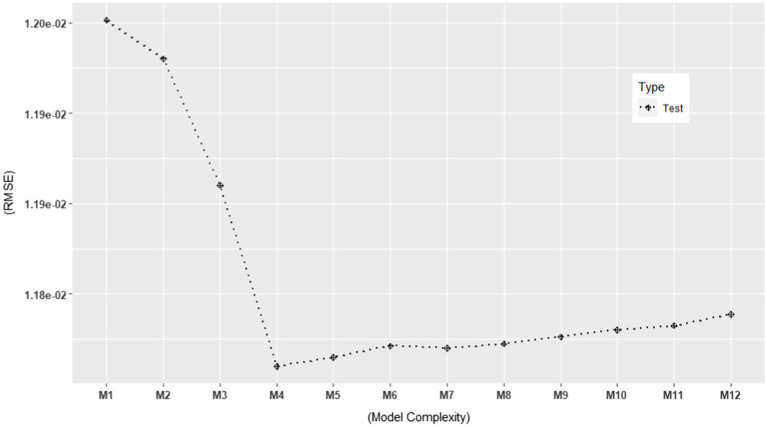
Test set RMSE for 12 models using the 2019 NSDUH data (*N =* 6,696). The RMSE values are consistent with those in Table 2.

Based on the ERA model in Kim and Hwang (2021), we constructed possible candidate models with different levels of model complexity. See Table 2 for the candidate models. As shown in the table, M4 represents the original ERA model. Our particular interest lied in selecting the best-performing model among the various candidate models. Thus, we estimated their performances using the model evaluation metrics discussed in the previous section in order to rank them against each other. More specifically, we computed the following model evaluation metrics using the NSDUH data from 2016 to 2018: (1) apparent (in-sample) RMSE, (2) ${\bar{\mathrm{Err}}}_{\left(\mathrm{cv,}10\right)}$, (3) ${\bar{\mathrm{Err}}}_{\left(\mathrm{OOB,}B=50\right)}$, and (4) ${\bar{\mathrm{Err}}}_{\left(.632,B=50\right)}$. In addition, we computed the models’ RMSE using the 2019 NSDUH data. The objectives of this investigation were 2-fold. First, it aimed to estimate the generalization performance of each model using the test set. Second, by plotting it, one could visually detect when overfitting started to occur. The results are summarized in Table 2, which are also graphically displayed in Figures 5, 6.

**Table 2:** Out-of-sample model performance metrics for the nicotine dependence ERA model using NSDUH data.

		${\bar{Err}}_{\left(cv,10\right)}$	${\bar{Err}}_{\left(OOB,B=50\right)}$	${\bar{Err}}_{\left(.632,B=50\right)}$	RMSE_(Train)_	RMSE_(Test)_
1	$y_{i}=b_{1}f_{i1}+b_{2}f_{i2}+e_{i}$	0.006624	0.006627	0.006626	0.006623	0.011961
2	$y_{i}=b_{2}f_{i2}+b_{3}f_{i3}+e_{i}$	0.006581	0.006587	0.006583	0.006579	0.011944
3	$y_{i}=b_{1}f_{i1}+b_{3}f_{i3}+e_{i}$	0.006547	0.006553	0.006548	0.006545	0.011888
4	$y_{i}=b_{1}f_{i1}+b_{2}f_{i2}+b_{3}f_{i3}+e_{i}$	0.006499	0.006505	0.006500	0.006496	0.011808
5	$y_{i}=b_{1}f_{i1}+b_{2}f_{i2}+b_{3}f_{i3}+b_{4}\left(f_{i1}\cdot f_{i2}\right)+e_{i}$	0.006501	0.006503	0.006497	0.006493	0.011812
6	$y_{i}=b_{1}f_{i1}+b_{2}f_{i2}+b_{3}f_{i3}+b_{5}\left(f_{i1}\cdot f_{i3}\right)+e_{i}$	0.006497	0.006501	0.006496	0.006489	0.011817
7	$y_{i}=b_{1}f_{i1}+b_{2}f_{i2}+b_{3}f_{i3}+b_{6}\left(f_{i2}\cdot f_{i3}\right)+e_{i}$	0.006494	0.006507	0.006497	0.006487	0.011816
8	$y_{i}=b_{1}f_{i1}+b_{2}f_{i2}+b_{3}f_{i3}+b_{4}\left(f_{i1}\cdot f_{i2}\right)+b_{6}\left(f_{i2}\cdot f_{i3}\right)+e_{i}$	0.006496	0.006507	0.006494	0.006484	0.011818
9	$y_{i}=b_{1}f_{i1}+b_{2}f_{i2}+b_{3}f_{i3}+b_{4}\left(f_{i1}\cdot f_{i2}\right)+b_{5}\left(f_{i1}\cdot f_{i3}\right)+e_{i}$	0.006498	0.006505	0.006494	0.006486	0.011821
10	$y_{i}=b_{1}f_{i1}+b_{2}f_{i2}+b_{3}f_{i3}+b_{5}\left(f_{i1}\cdot f_{i3}\right)+b_{6}\left(f_{i2}\cdot f_{i3}\right)+e_{i}$	0.006495	0.006504	0.006492	0.006480	0.011824
11	$y_{i}=b_{1}f_{i1}+b_{2}f_{i2}+b_{3}f_{i3}+b_{4}\left(f_{i1}\cdot f_{i2}\right)+b_{5}\left(f_{i1}\cdot f_{i3}\right)+b_{6}\left(f_{i2}\cdot f_{i3}\right)+e_{i}$	0.006496	0.006509	0.006492	0.006477	0.011826
12	$y_{i}=b_{1}f_{i1}+b_{2}f_{i2}+b_{3}f_{i3}+b_{4}\left(f_{i1}\cdot f_{i2}\right)+b_{5}\left(f_{i1}\cdot f_{i3}\right)+b_{6}\left(f_{i2}\cdot f_{i3}\right)+b_{7}\left(f_{i1}\cdot f_{i2}\cdot f_{i3}\right)+e_{i}$	0.006502	0.006526	0.006498	0.006469	0.011831

In Figures 5, 6, we plotted the 12 candidate models on the x-axis, from simple to complex models, against the RMSE values on the y-axis. In Figure 5, the apparent RMSE shows a typical learning curve: The model performance on the training set consistently decreases with model complexity. By looking at the test set RMSE in Figure 6, where the generalization error goes down only until a turning point M4, we can clearly see M5 to M12 overfit the training data. Interestingly, all the out-of-sample model evaluation metrics did not show the same pattern as the apparent RMSE: after M4, they almost stopped decreasing with increasing model complexity. Moreover, their error trend even changed from descending to ascending after M10. In addition, all of them alleviated the optimism in the apparent RMSE. These findings support the simulation results that the out-of-sample metrics are more likely to avoid overfitted models than the traditional in-sample metrics for ERA. To build a model that can generalize to unseen samples, especially when overfitting is of concern, the use of the out-of-sample model assessment metrics is crucial in model selection.

## Concluding Remarks

In many psychology studies, it is often assumed that a sample at hand (i.e., a training set of data) is a good reflection of what will be encountered in the future; thus, the final model is selected as the one optimized for the training data (Shmueli, 2010; Bryant, 2016). It is not ideal to compare candidate models based on such in-sample GOF assessment because GOF model evaluation metrics always favor more complex models (which fit the training data too tightly), thereby limiting the generalizability of the selected model. But estimating a model’s future performance only using information contained in the current sample is also a hard problem. To respond to this, we discussed the use of computer-intensive resampling methods, including variants of CV and the bootstrap, in order to provide new ways of assessing generalizability of ERA models. There has been no discussion in the ERA literature as to out-of-sample error estimation for model selection, and no comparison of widely used resampling methods has been conducted to date. Thus, we discussed the predictive modeling of ERA in the context of model selection, particularly the derivation and evaluation of new model selection criteria.

Our simulation study showed that the optimism of conventional in-sample model evaluation metrics was negligible in large samples (e.g., *N* ≥ 500) but never disappeared. This implies that comparing candidate models based only on conventional FIT or in-sample RMSE is not recommended because these model evaluation metrics always favor more complex models, thereby being unable to select a model that results in a reproducible conclusion for future data. The simulation study also highlighted the advantage of adopting CV and bootstrap methods to avoid overly optimistic assessment of a model’s predictive performance. Over a wide range of different sample sizes and model complexities under correctly and incorrectly specified model conditions, all of the out-of-sample prediction error estimators favored the true model with the highest frequency. In fact, owing to improvements in statistical computing over the past years, it has become substantially easier to execute various resampling methods on modern laptops without much computational burden.

The study’s general conclusions may be summarized as follows. Firstly, the our-of-sample model evaluation metrics based on 10-fold CV, 0.632, and 0.632+ methods outperformed other resampling strategies, but the differences between them became unnoticeable in large samples, e.g., *N* ≥ 200, even for mis-specified models. Secondly, when highly complex models are considered, the *c*-fold CV with a small number of folds and the regular OOB bootstrap methods may perform poorly compared to other resampling methods. Thirdly, for largely over-specified models, the LOOCV estimator was a reasonable choice as it resulted in the lowest bias and variability. Fourthly, *B* = 20 bootstrap replications would be sufficient for the regular OOB bootstrap estimator and its variants. The advantage of increasing *B* from 20 to 100 was minimal in terms of the variability of estimators. Lastly, overall, the 0.632 and 0.632+ estimators outperformed other estimators, but the 10-fold CV prediction error estimate approximated those of 0.632 and 0.632+ in almost all settings. Thus, for computationally burdensome analyses, 10-fold CV may be preferable over the OOB bootstrap estimators.

As the first initiative in investigating out-of-sample model performance of ERA, the broader goal of this study is to bring this discussion into the field of psychology so that such predictive model assessment metrics can be effectively utilized for investigation of reproducibility and generalizability of psychological and behavioral data science. Thus, we also demonstrated how the proposed model evaluation metrics could be utilized in practice, using a well-known national survey data set in the US. The out-of-sample model evaluation metrics did not show the continuously decreasing trend of the conventional in-sample metric. This suggests that the out-of-sample metrics are reliable in avoiding overfitted models and can be utilized as a tool to estimate the generalization error in the absence of a large independent test set. Using out-of-sample methodological practices to access a model’s predictive performance received little attention in the psychology literature, however, it comes to be applied more frequently in recent years, especially with the reliance on CV (e.g., Yarkoni and Westfall, 2017; Koul et al., 2018; de Rooij and Weeda, 2020).

In this paper, we discussed the assessment of prediction performance in terms of RMSE. Thus, the simulation results can be widely applicable for other ERA models for continuous responses fit by expected squared-error loss. However, when response variables are discrete—which is often termed *classification problems* in statistics and machine learning, the best choices of resampling methods may differ substantially. Simulation studies on prediction error estimation in classification problems (e.g., Efron, 1979; Hastie et al., 2009, Chapter 7.3) demonstrate that the bias and variance of expected test error in (7) behave considerably differently for classification loss functions (e.g., 0–1 loss, the negative binomial log-likelihood known as *deviance* or *cross-entropy*) than they do for squared-error loss. Thus, optimistic estimation of model performance in classification problems should be further examined. A variety of loss functions for prediction error need to be considered. Especially, understanding the effect of the number of events per variable (EPV), instead of simple sample sizes, is important when binary or multinomial response variables are considered.

## Data and Code Availability

Original NSDUH data are openly available in the Substance Abuse and Mental Health Data Archive (SAMHDA) at https://datafiles.samhsa.gov/. The aggregated data used in Empirical Application are available at https://github.com/QuantMM/PredictiveERAmodeling. Simulation source code is also available at the same GitHub page.

## Data Availability Statement

Publicly available datasets were analyzed in this study. This data can be found at: https://www.datafiles.samhsa.gov/.

## Author Contributions

SK contributed to conception and methodology, organized the data, performed the simulation and statistical analysis, and wrote and edit the original draft. HH read the draft and contributed to manuscript revision. All authors contributed to the article and approved the submitted version.

## Funding

This work was supported in part by University of Manitoba’s University Research Grants Program (project no. 54366) awarded to SK.

## Conflict of Interest

The authors declare that the research was conducted in the absence of any commercial or financial relationships that could be construed as a potential conflict of interest.

## Publisher’s Note

All claims expressed in this article are solely those of the authors and do not necessarily represent those of their affiliated organizations, or those of the publisher, the editors and the reviewers. Any product that may be evaluated in this article, or claim that may be made by its manufacturer, is not guaranteed or endorsed by the publisher.
